# Mingled bodies and voices: Maternal reflections on caregiver expertise and intellectual disability

**DOI:** 10.1177/17446295211009339

**Published:** 2021-05-10

**Authors:** Laura MacGregor

**Affiliations:** Wilfrid Laurier University, Canada

**Keywords:** family caregiving, intellectual disability, COVID-19, intersubjectivity, essential expertise

## Abstract

The risk of viral infection during the COVID-19 pandemic has caused many hospitals to prohibit all patient visitors, including family caregivers for people with intellectual disabilities. Drawing on a postmodern, intersubjective view of the body, as well as my experience as the mother of a young adult with profound disabilities, I argue that caregiver knowledge while unconventional within the medical paradigm must be viewed as essential expertise. People with profound intellectual disabilities often have concurrent, complex medical issues that are complicated by their inability to self-advocate. Optimal care rests upon the ongoing presence and expertise of their primary caregiver. Medical professionals risk patient care by excluding the essential expertise of family caregivers at any time, and specifically during COVID-19.

On March 15, 2 days into our Canadian province’s COVID-induced state of emergency, my 21-year-old, profoundly disabled, non-verbal, medically fragile son was ambulanced to hospital with breathing difficulties and a fever. Upon arriving to the emergency room, I sat in a negative air pressure room wearing full PPE and watched his oxygen saturation plummet. A few hours later my son, Matthew, was on a ventilator in the intensive care unit of our local hospital. Matthew did not have COVID-19, simply aspiration pneumonia. But because of the timing and nature of his admission, COVID-19 strongly influenced his admission and care.

Four days after Matthew’s admission, as confirmed COVID-19 patients began to fill the ICU, hospitals in our province adopted a universal ‘no-visitors’ policy. Exceptions were made for palliative care, but all outsiders including parents and primary caregivers for patients with intellectual disabilities, were routinely barred from their loved-one’s bedside. A hospital administrator was dispatched to my son’s ICU room where I was told that ‘visiting’ was no longer allowed. I was to leave the hospital and my return would be considered on the day Matthew was to be extubated.

The hospital’s concern about managing the community spread of COVID-19 was understandable, and strict limits on visits were, and remain, a prudent response to a very serious health crisis. However what the new visitor policy failed to acknowledge was that as the mother and lifelong caregiver for my son living with intellectual disabilities, my unique knowledge was both inaccessible to health care providers operating within the context of the medical model, and indispensible to providing optimal care. Preventing access to my son during his hospitalization would compromise his care. I vigorously challenged the policy.

While evolving, medical care tends to view the human body through a reductionist lens (Mehta, 2011), and knowledge is oriented around the scientific method (Lincoln and Guba, 1985). The grounding of health care within a Cartesian, positivist paradigm poses unique challenges, particularly for caregivers of dependent individuals who live with very severe intellectual disabilities. Drawing on a postmodern understanding of a complex and porous body, as well as my journey as Matthew’s mother, I will argue that when caring for people with profound intellectual disabilities the intuitive, intersubjective knowledge between the caregiver and individual with intellectual disabilities, while unconventional within the paradigm of the medical community, is crucial to optimal care. Correspondingly, as Matthew’s mother I was not a visitor, I was an expert able to glean unique and indispensible insight into my son’s body that was necessary for his care.

## Matthew and me

At the time of Matthew’s admission I was the mother of three young adult men aged, 19, 21, and 23. Matthew, my middle son, lived with a complex list of disabilities and medical challenges subsequent to a severe birth injury. Specifically, Matthew lived with cerebral palsy, profound intellectual disability, seizures, reflux, a feeding tube, and respiratory difficulties. Matthew did not speak and relied on a team of caregivers to anticipate and respond to his needs. Yet despite his numerous challenges, Matthew lived a rich life. He loved time at our family cottage, bubbles, music, colourful cartoons, and was an avid fan of the Kitchener Rangers, our local hockey team.

For the first decade of Matthew’s life I provided his full-time care with little respite or outside support. It was only once his care needs exceeded my personal abilities that I assembled a team of caregivers to assist me. My years of sustained and intense caregiving provided the foundation for an intimate knowledge of my son’s body and medical status. As a result I had an uncanny ability to ‘read’ my son’s body and non-verbal cues, and could seamlessly integrate emerging information with his complex medical history.

Initially my background as an occupational therapist assisted my ability to care for Matthew. My training as a health professional supported my ability to organize environmental adaptations, address Matthew’s mobility needs, and navigate the dizzying array of health care providers. However as Matthew’s complex medical status evolved, like all parents my expertise adapted to his unique challenges. I learned to manage seizures, a feeding tube, and assess complex non-verbal pain and respiratory distress.

While my training in the health sciences no doubt provided a solid foundation, as Matthew’s mother I developed an intuitive ability to interpret my son’s unique and non-verbal communication that did not translate well within the medical paradigm. This unorthodox knowledge informed accurate predictions about my son’s overall health and medical status; I could often correctly predict the outcomes of clinical assessments. Yet, I was frequently frustrated and surprised that despite my fluency in medical jargon, and the accuracy with which I interpreted my son’s cues, the medical team frequently dismissed my maternal wisdom. This ongoing challenge motivated me to pursue graduate work in the area of caregiving.

## Navigating medical care

Until the early 1960s the deficit based, reductionist medical model dominated all medical conversations. In recent decades, particularly in light of the World Health Organization’s (2001) International Classification of Functioning, Disability, and Health, illness and disability are increasingly understood as a complex interplay of human embodiment amid vast array of social, environmental, and cultural influences. In response to this evolution in medical thinking, educational programmes for medical and health professionals increasingly emphasize a collaborative approach to care. Parents and family members are viewed as integral and necessary members of the health care team, though in clinical settings practical application of this ideal remains inconsistent (Olding et al., 2016).

Mirroring the research, the inclusion of my knowledge regarding Matthew’s health varied greatly among health care environments. While some teams enthusiastically included me in all aspects of Matthew’s care, other professionals viewed my maternal expertise with skepticism, or outright dismissal. Research suggests my experience was not uncommon among parents of children with significant disabilities (Ryan and Quinlan, 2017; Todd and Jones, 2003; Woodgate et al., 2017). I can provide numerous examples when my son’s medical care was compromised because a physician was reluctant to share assessment and decision-making power with a mother. At worst, I was labelled a hysterical, helicopter mother.

While there is no doubt that the medical paradigm is evolving in a more holistic direction, two philosophical ideas continue to inform medical practice. First, the body is viewed primarily through Cartesian dualism, and therefore a reductionist, lens (Mehta, 2011). And second positivism, particularly with respect to the notion of knowledge as supported by the scientific method, defines what constitutes as acceptable information (Branson, 1998; Solomon, 2015).

In 1637 Rene Descartes published his influential book *Meditations and Other Metaphysical Writings* (Descartes, 1998). In this book, Descartes emphasized a separation between a mechanistic body and the vastly superior mind. By extension, since the body itself was divisible, bodies were bounded, discrete entities. Porousness within the body was suspect, and between bodies was unthinkable.

Positivism continues to dominate scientific thought, and therefore medical practice. The positivist paradigm, among other ideals, assumes that reality is tangible, the knower and known are separate and therefore dualistic, and that research is value-free and can be generalized. It is understood that science is engaged a never-ending quest to determine a specific truth, indeed positivism rests upon the belief that there is a single, tangible reality that can be identified and known (Lincoln and Guba, 1985).

Embracing positivism, medical practice is founded on the assumption that knowledge is generated and validated through evidence-based study (Branson, 1998; Solomon, 2015). Solomon (2015) argued, ‘Evidence-based medicine de-emphasises intuition, unsystematic clinical experience and pathophysiological rationale as sufficient grounds for clinical decision-making and stresses the examination of evidence from clinical research’ (p. 106). Clinical researchers argue that data are valid, reliable, replicable, and unbiased (Lincoln and Guba, 1985). As a result, medical care understands the body through measurable, observable, and objective criteria. Maternal and caregiver knowledge functions outside these parameters, and is therefore suspect.

As a former health science student educated during the late 1980s, I was thoroughly steeped in the medical model and scientific method. I value the corresponding advancements in medicine, and was relieved when the medical paradigm quickly and accurately identified and treated my son’s health care challenges. However because my son lived with complex and multiple conditions, and could not self-advocate, his care was complicated. Assessment of his clinical presentation was heavily nuanced and relied on an intimate understanding of his body considered against the backdrop of his lengthy medical history. Because he lived with a significant intellectual disability and was non-verbal, standard clinical assessments were often ineffective in isolating pain or identifying the root of a medical crisis without my guidance. These were moments when my intuitive understanding of his body and medical status was most important.

Unfortunately, this unorthodox ‘knowing’ of my son’s body presented epistemological challenges for a profession firmly entrenched in a very specific, and power-laden way of defining knowledge. My inability to offer evidence that met the necessary criteria of medical knowledge was an ongoing challenge when navigating health care environments. The patriarchal world of medicine has the power to define what constitutes valid knowledge, and my maternal wisdom was at times dismissed as subjective and unreliable, often compromising my son’s care. In contrast, improved care ensued when medical professionals were comfortable sharing power and valuing unconventional knowledge.

## Leaky, porous bodies

Several years ago I advocated to my son’s physician for improved pain control. Over the counter medications no longer appeared to be controlling his chronic discomfort and I requested a stronger PRN medication. At the time Matthew’s pain was poorly understood, and as a result the physician was reluctant to prescribe medication without understanding the cause. Frustrated with the physician’s rationale I argued that his inability to diagnose my son’s pain should not be the cause of Matthew’s suffering. As the physician turned to leave the room he handed me a prescription for a narcotic and said, ‘perhaps by treating your son I will calm *you* down’. This sentence would become the foundation of my doctoral work, and continues to be the source of ongoing personal reflection.

The physician’s dismissal of my son’s suffering aside, what fascinated me about this encounter was that the physician, with a single sentence, challenged a Cartesian understanding of separate bodies and suggested that our bodies were interconnected; that by treating my son’s body he could impact my body. Following this encounter I began to question how maternal-child intersubjectivity might inform maternal knowledge and influence a mother’s care and advocacy. As we consider the role of parental expertise during COVID care, I think this postmodern view of the body is relevant.

According to Shildrick (1997) a postmodern critique of the body assumed the following: (a) a coherent understanding of truth associated with the enlightenment was replaced by a fragmented approach to knowledge; (b) because this unified understanding of the world had passed, grand narratives including medical science’s understanding of the body, must be abandoned in favour of a fragmented and problematized understanding, and finally (c) boundaries between discrete bodies of knowledge were challenged, complicating what constitutes knowledge, particularly in terms of the distinction between theory and the lived experience. Taken together, these assertions strongly favour an abandonment of an exclusively reductionist embodiment of traditional Western medicine (Cregan, 2012), and favour a holistic, nuanced, relational, and interconnected understanding of the body (Goodley and Runswick-Cole, 2013; Shildrick, 1997, 2009). Such a postmodern intersubjective view of the body is particularly relevant for people living with profound intellectual disabilities who rely on caregivers to understand their body, and advocate for their health care needs.

Philosopher Merleau-Ponty (2010) first argued that objects could extend the boundaries of the body. For example, he suggested that a person living with a visual impairment used a white cane as an extension of their body. The cane became part of the body, and like fingertips, provided rich sensory information. Emerging research in the field of caregiving supported this problematized body. Rather than the dualism of caregiver and cared-for as bodies with sharply defined boundaries, bodies were viewed as complex assemblages intersecting with machines (wheelchairs and feedings tubes), animals (seeing-eye dogs), and people (partners, friends, caregivers, lovers) (Churchill, 2012; Fritsch, 2010; Gibson, 2006; Price and Shildrick, 2002).

My son’s body was not limited by his skin, but encompassed his wheelchair, feeding tube, as well as another body, me his caregiver, who could interpret his body and give voice his experiences. While there is little research exploring interconnection and care, a handful of qualitative studies including my own doctoral dissertation suggested that experiences between intimately connected caregivers and care receivers were leaky, with the feelings and actions of one wordlessly informing the other (Churchill, 2012; Fritsch, 2010; Gibson, 2006; MacGregor, 2019, Price and Shildrick, 2002). In particular, ethnographic research by Goodley and Runswick-Cole (2013) argued that deeply relational, complex, disabled bodies disrupted the culturally enforced, normative view of bodies as discrete and autonomous. This postmodern notion of disrupted, complicated, extended, and leaky bodies is the foundation of caregivers’ ability to ‘read’ their loved-one’s body and advocate for their needs.

## Mingled voices and bodies: Balancing the ethical challenges

While this notion of complex, interconnected embodiment helps us unpack how mothers and caregivers might have access to unique knowledge about their family members, a postmodern view of the body poses legitimate ethical questions. When mothers speak on behalf of their children with profound intellectual disabilities it can be challenging for professionals to discern voice, power, and authorship. Swinton et al. (2011) noted that the mingling of voices is particularly concerning for people who may not be able to communicate their wants and needs. People with intellectual disabilities risk becoming ‘victims of constructions of their stories that they do not own’ (p. 6). Medical researchers have echoed such concerns about working with parents who speak on behalf of their child with intellectual disabilities, particularly with respect to voice and agency (Woodgate et al., 2017). It is imperative that people with intellectual disabilities are provided with the means to self-advocate whenever possible. However there will always be a small population of completely dependent people who require the support and advocacy of their caregivers and it is essential that health care providers understand this enmeshed dyad. Further research exploring interconnection, intellectual disability, power, voice, and advocacy is required.

As the mother of a child with disabilities, and an ally of people living with disabilities, I am sympathetic to these ethical concerns. Self-advocates have rightly noted that society has a long history of silencing people with disabilities, and this has meant that the voices, rights, needs, power, and desires of people with disabilities have been oppressed (Charlton, 1998). However, the disability rights movement has been primarily influenced by the concerns and needs of people living with physical disabilities. The concerns and challenges experienced by people living with intellectual disabilities have not received the same attention. Many people with intellectual disabilities rely, to some extent, on caregivers to identify and advocate for their needs. And some like my son may be completely dependent. Dismissing the needs of this small and very vulnerable segment of our communities, as well as the expertise of their longstanding caregivers, poses significant risks and is nothing short of intellectual ableism.

## Caregivers are essential health care experts

Prohibiting visitors during the COVID pandemic, while distressing, makes sense when patients are able to self-advocate. The balance of risks tips precariously towards the negative when caregivers are barred from the acute care of their loved one living with an intellectual disability. Four days into Matthew’s ICU admission, a hospital administrator informed me that due to COVID-induced restrictions, I could no longer ‘visit my son’. According to the hospital administration, the risk of my presence in the hospital outweighed the risks associated with my absence. That this balance of risks did not consider my son’s unique embodiment and needs was evident. Telephone calls were briefly discussed as a means of including me in decision-making, but this would mean that I was relying on the information of an outsider who did not have the same ‘sense’ of my son when considering care decisions. I did not accept this alternative.

During my conversations with the hospital administration I argued that labelling my time with Matthew as ‘visiting’ was erroneous. I was not spending time in the hospital for social reasons. Rather, I was spending time with my son to monitor his ongoing health. I suggested that I possessed unique expertise essential for providing optimal care, and as a result my movement between community and hospital should be managed similar to health care professionals working in the hospital.

I must make clear that I don’t wish to minimize the value of medical expertise. As a researcher, I value evidence-based practice. However, I also assert that the unique and unconventional knowledge of caregivers constitutes necessary expertise when caring for complex and vulnerable patients, particularly those with significant intellectual disabilities who are unable to self-advocate. My interconnection with my son supported my ability to interpret his body and use this information to advocate for his care. That this knowledge was inaccessible to the health care professionals operating within the traditional medical model further highlighted its relevance, particularly in light of Matthew’s complex embodiment and medical status. I must emphasize that I am not proposing an either/or dichotomy between the scientific method and caregiver knowledge, but rather both/and. Caregivers and health care professionals must avoid competitive relationships. Relationships that share power and value diverse knowledge are essential.

## Concluding thoughts

Fortunately, my advocacy efforts proved successful. Throughout my son’s hospitalization my presence in the hospital was permitted and I continued to be included in all health care conversations. Initially there was great hope, even an expectation that my son would do well. Matthew had a longstanding history of recovering from pneumonia, and the entire ICU team expected this hospitalization would be no different from previous ones. Sadly Matthew’s lungs were badly compromised from a lifetime of respiratory challenges, and as the days passed it become increasingly clear that this admission was different. My involvement and expertise became increasingly necessary during ICU team discussions, particularly as the team compared this hospitalization to my son’s previous illnesses and we collectively grappled with ethical concerns regarding reasonable quality of life and survivability. After 14 days on a ventilator, aggressive intervention was replaced with palliative care. Matthew passed away a few hours later.

COVID-19 has highlighted numerous gaps in care for people with disabilities, including the imperative for people with intellectual disabilities to have access to their most reliable advocates during acute care. Matthew’s hospitalization occurred at the outset of the pandemic during a time when the public discourse in Canada and North America was concerned with the ethics of triaging of scarce resources, particularly with respect to people with disabilities. Since Matthew was admitted to hospital 2 days into our province’s state of emergency he was likely one of the first patients with a severe intellectual disability to challenge the newly imposed restrictions on family caregivers in acute care settings. In the months following my son’s admission provincial disability advocates identified the risks for people with intellectual disabilities, vigorously challenged the policy restricting family caregivers, and developed toolkits for families and people with disabilities (for example see Arch Disability Law Centre, 2021).

The prevailing view during this early stage of the pandemic was that all family members constituted visitors, and that all forms of visitation were prohibited outside of palliative admissions. In order to ensure my son’s best care it was incumbent upon me to challenge senior health officials in the hospital and persuasively advocate for my continued presence at my critically ill son’s bedside. This imperative for family caregivers to navigate bureaucratic structures and challenge those who hold power to ensure their loved one’s optimal care is overwhelmingly burdensome. Indeed, work by Goodley and Runswick Cole (2011) would suggest that such systemic structures that impede the best care for people with disabilities constitute disablism and are a form of violence.

Hospitals must acknowledge that when caring for people with significant intellectual disabilities family caregivers are not visitors, but are essential members of the health care team. Their expertise, while unorthodox, is both necessary for optimal care and often inaccessible to health professionals operating within scientific paradigm. Barring family caregivers during COVID-19 hospitalizations is discriminatory, and seriously risks effective patient care for people with profound intellectual disabilities. Inherent to acknowledging parental/caregiver expertise is the need for medical providers to share power, acknowledge that valuable expertise can evolve outside of the traditional scientific method, and embrace a more collaborative approach that values diverse contributions to medical conversations. Hospital policies, at all times, including during the COVID-19 pandemic, must acknowledge the essential expertise and contributions of caregivers for people with significant intellectual disabilities. Further, family members must not be placed in the difficult position of challenging powerful professionals and bureaucracies to ensure their loved-ones’ best care. Strategies to ensure the safe and consistent presence of caregivers in hospital are essential for optimal care and policies must be established to ensure the stable presence of advocates for people with intellectual disabilities during all hospitalizations.
